# “It’s beyond enough”: teachers’ experiences and perspectives of rehabilitation inclusion for working with deaf and hard of hearing children in rural areas of China

**DOI:** 10.1186/s12889-025-22972-1

**Published:** 2025-05-19

**Authors:** Zhizhuo Wang, Wangwei Lin, Hong Qiu, Zebo Lan

**Affiliations:** 1 School of Health, Fujian Medical University, No. 1 Xuefu North Road, University Town, Fuzhou, Fujian 350122 China; 2 Nanping School for the Deaf-Mute, Jian’ou No.149, Daxi Alley, Nanping, Fujian 353100 China

**Keywords:** Deaf and hard of hearing children, Teachers, Rehabilitation inclusion, Qualitative study

## Abstract

**Background:**

Evidence suggests that individuals who are deaf and hard of hearing (DHH) are a heterogeneous population and may present with their unique needs that set them apart from general disability group. Current studies have focused on the parents’ experiences of DHH, lacking evidence on teachers’ experiences and perspectives of rehabilitation inclusion in Chinese rural context. Thus, this study aimed to identify key themes from teachers’ experiences regarding the challenges (“bad” things) and successes (“good” things) in educating students with DHH, while also exploring their perspectives of rehabilitation inclusion within the context of the Chinese educational system, particularly in rural areas.

**Methods:**

The exploratory qualitative research design was employed in this study. Participants were recruited from a public special education school situated in Jian’ou, Fujian province, China by using a purposive sampling strategy. Interviews were audio-recorded, transcribed verbatim, and analyzed using Braun and Clarke’s thematic analysis.

**Results:**

After the nineteenth interview, data saturation was deemed achieved. Five themes emerged from the collected data analysis: (1) role as a life-and-study co-instructor, (2) teacher-and-student bidirectional social, emotional, and financial support, (3) diverse and inclusive communication between teachers and deaf and hard of hearing children, (4) lack of policy-related, institutional and cultural information about deaf and hard of hearing children, and (5) awareness the rehabilitation to be integrated within the educational context.

**Conclusion:**

The findings suggested that more attention should be focused on public special education school in underdeveloped rural areas of China. Although this study provided rich information of the targeted population, future research should incorporate rigorous quantitative methods to strengthen the scientific foundation of the findings and enhance their generalizability.

**Supplementary Information:**

The online version contains supplementary material available at 10.1186/s12889-025-22972-1.

## Background

According to the World Health Organization (WHO), nearly 20% of the global population experiences some degree of hearing loss, with 430 million currently living with disabling hearing loss. By 2050, the number of individuals with disabled hearing loss could rise to over 700 million [1]. In China, there are over 20 million individuals with hearing disabilities, including both children and adults [2]. These figures highlight the growing public health challenges posed by hearing impairment worldwide. Within this context, it is essential to clarify the terminology used to describe hearing loss. The term “deaf” typically refers to individuals with severe to profound hearing loss, characterized by slight or no functional hearing. In contrast, “hard of hearing” describes those with mild to moderate hearing loss, where auditory devices, such as hearing aids, can significantly compensate for the impairment [3]. In this article, “deaf and hard of hearing” (DHH) is used inclusively to encompass both groups, reflecting the diverse spectrum of hearing loss and its varying impacts on individuals.

In China, children with deaf and hard of hearing problems in rural areas are often enrolled in government-funded special education schools. The phenomenon may be largely or partially explained by the unique needs of children with DHH, who represent a heterogeneous population that sets them apart from other disability groups. These distinct needs include the development of auditory or sign language skills (or both), the cultivation of cultural identity awareness and acceptance, the usage and troubleshooting of hearing devices (if accessible), and the acquisition of self-advocacy skills [4]. Such specialized needs often necessitate tailored educational environments and support systems, which general education settings may not adequately provide. While in the special education schools, a team of experienced professionals and paraprofessionals (primarily teachers in the special education schools) can deliver tailored services to students with DHH, such as designing and implementing appropriate communication and learning opportunities to meet their unique needs [5]. For children with DHH, the transition to school often comes with heightened demands or expectations, such as elevated requirement for routine obeyance, relatively longer participation in variety of curricular or extracurricular activities, navigation among teacher and peer interactions [6]. In addition to these challenges faced by children with DHH, teachers, as the primary point of contact and interaction for these children, also encounter significant difficulties. This is particularly true in rural areas of China, where students with disabilities often travel from remote regions to attend school, separating from their parents and familiar environments for extended period. Therefore, understanding the teachers’ experiences for working with students with DHH can provide invaluable insights and inform the development of more effective educational practices in the underserved settings.

By searching the database about relevant topics of children with DHH, we found that the majority of research has predominantly focused on the parental experiences. For example, a comprehensive review including 111 children, 23 families and 41 parents highlighted the unique familial challenges faced by parents of DHH children [7]. Although one study explored the experiences and insights of teachers and educators, it specifically addressed deaf-blind learners, a population distinct from the DHH individuals [8]. Deaf blindness, characterized by a dual sensory impairment affecting both vision and hearing [9], is a relatively rare condition affecting approximately 1 million individuals globally [8]. Additionally, a fieldwork-based study conducted at Anushruti Academy for the Deaf in India identified emerging themes from the lived experiences of teachers, including issues of language and speech acquisition, methods of teaching, dialectical relationship of students to technology, and experiences of stigma [10]. While these studies have provided valuable insights into the experiences of parents and teachers in specific contexts, the experiences of teachers in Chinese rural areas remain underexplored.

Early rehabilitation is widely recognized as critical for the development of students with DHH. Evidence suggests that early and timely intervention, combined with tailored hearing technology, can enhance neural plasticity during the early developmental stages, potentially preventing or mitigating the risk of impairments in brain maturation [11]. Early rehabilitation programs for DHH students typically adopt a multidisciplinary approach. First and foremost, the foundation of early rehabilitation is timely identification of hearing loss, often through newborn hearing screening programs [12]. Second, the use of hearing aids or cochlear implants, combined with auditory training, is a cornerstone of early rehabilitation [13]. Third, speech-language pathologists play a central role in helping DHH children develop communication skills. Therapy may focus on spoken language, sign language, or a combination of both, depending on the child’s needs and family preferences [14]. Fourth, programs often include training for parents to help them support their child’s development at home because families are integral to the success of early rehabilitation [15]. Lastly and importantly, early rehabilitation programs often include counseling and social skills training to address the emotional and social challenges faced by DHH children and their families [16]. However, awareness of its importance is often limited among both teachers and students. Teachers play a pivotal role as intermediaries between students and their parents or guardians, making their understanding and implementation of rehabilitation practices essential. It is therefore crucial to investigate whether teachers recognize the value of rehabilitation and the extent to which they incorporate it into their educational practices.

Taken together, this study aimed to identify key themes from teachers’ experiences regarding the challenges (“bad” things) and successes (“good” things) in educating students with DHH, while also exploring their perspectives of rehabilitation inclusion within the context of the Chinese educational system, particularly in rural areas. By addressing this gap, the study seeks to provide valuable insights and potentially inform reforms in the Chinese special education system.

## Methods

### Research design

The interpretive qualitative research paradigm was employed in this study to explore the teachers’ experiences and perspectives of rehabilitation inclusion for working with deaf and hard of hearing children in rural areas of Fujian province. By adopting an interpretive approach, the study can capture the rich, context-specific insights that are essential for understanding the complexities of the teachers’ roles and experiences [17]. The study was guided by the Consolidated Criteria for Reporting Qualitative Research (COREQ) checklist [18].

### Participants and context

A purposive sampling strategy was employed to recruit teachers from a public special education school in Jian’ou, Fujian province, China, during the period of January to February 2024. Teachers who met the following criteria would be considered for recruitment: (1) had already obtained teacher qualification certificate for teaching students with disabilities; (2) had more than one-year teaching experiences with students who are deaf and hard of hearing; (3) had normal hearing capabilities for interview. The public special education school serves students with a range of disabilities including DHH, from diverse rural areas in northern Fujian Province, providing them with access to tailored educational opportunities. Northern Fujian Province, known as Minbei in Mandarin, is situated in the northern part of Fujian Province, China. This region is characterized by its predominantly rural landscape and relatively underdeveloped economy, particularly when compared to the more prosperous coastal and urban areas in southern Fujian (e.g., Xiamen). As such, the education system development in rural areas has significantly lagged behind that of urban areas [19]. This context underscores the unique challenges faced by teachers in providing resources and support for students with DHH, which makes it a unique setting for exploring the experiences of teachers for working in the public special education school.

The public special education school integrates three-year preschool education (speech therapy for deaf and hard of hearing children), nine-year compulsory education, and three-year vocational high school education. Based on the recent data, the school accommodates more than one hundred and thirty students with disabilities, among whom students with DHH comprise approximately 20 ~ 40%. The school covers an area of 20,000 square meters approximately, with standardized plastic playground, a comprehensive teaching building, a vocational education building, dormitory buildings, a canteen and other essential infrastructure. Guided by the educational philosophy of “people-oriented, scientific education, overcoming barriers, and diversified development”, the school has actively promoted inclusive education, implemented home-based education initiatives, and early rehabilitation (primarily the preschool language and speech training conducted by qualified professionals) to meet the diverse needs of its students.

### Procedures and data collection

Potential participants were initially identified based on their direct experience in teaching students with DHH and their willingness to share their perspectives. The research team contacted eligible teachers through school administrators, to explain the study’s purpose and procedures. Those who expressed interest were provided with a detailed information packet outlining the study’s background, aims, procedures, potential benefits, and risks, ensuring they were fully informed before participation. Prior to the commencement of semi-structure interview, interviewees would reiterate the study information briefly. This step was crucial to confirm their comprehension and willingness to proceed. Oral consent for audio recording during the interview was then obtained. Afterward, the audio-recorded data were transcribed verbatim, followed by returning the transcripts to participants for clarification and confirmation. The semi-structured interview questionnaire was developed by the research team, followed by a consultation in five teachers. The questionnaire was finalized by addressing the comments emerged in the consultation. The sample interview questions as shown in the following (refer to Supplementary file 1 for details): (1) Could you tell me about your experiences with deaf and hard of hearing children? (2) How do you communicate with deaf and hard of hearing children? (3) Could you tell me about your relationship with deaf and hard of hearing children?

### Data analysis

Data analysis was performed by the two authors according to Braun and Clarke’s thematic analysis procedure [20]. Inductive thematic analysis is a qualitative descriptive approach to identify, analyze, and summarize patterns (themes) within textual data [21]. The process began with two researchers (ZW and WL) independently reading the entire text multiple times to familiarize themselves with the content. They then held virtual or in-person meetings to discuss their overall understanding to reach a consensus on the core meaning of the data. Next, preliminary codes were developed by manually categorizing excerpts from the text. These codes were further redefined into potential themes, supported by relevant quotations as evidence. Subsequently, two additional researchers (HQ and ZL) reviewed the identified themes to evaluate their validity. The similarities and differences in the findings between the first two researchers were discussed among the four researchers to reach a consensus on the identified themes. To enhance the credibility of the findings, the identified themes were returned to the participants for validation to finalize the themes. Lastly, a comprehensive report was produced to present the thematic findings.

### Rigor and trustworthiness

Lincoln and Guba’s criteria including credibility, transferability, dependability and confirmability were employed in this study to secure the rigor and trustworthiness of this study [22]. Credibility was ensured through the following strategies: (1) prolonged engagement with participants to build trust, establish rapport, and clarify their responses; (2) member checking where participants were invited to review and provide feedback on emerging interpretations to ensure accuracy; and (3) peer debriefing involving discussions with colleagues to critically examine and validate various aspects of the research process. Transferability was supported by providing a thick description of the study context, participant characteristics, and observed experiences and processes. Dependability and confirmability were achieved through a comprehensive inquiry audit trail.

### Reflexivity

Reflexivity refers to the critical and ongoing examination of the researchers’ role, backgrounds, perspectives within the qualitative research process [23]. The research team consisted of two doctoral researchers with background in rehabilitation and education, and two school administrators in the public special education school. Given the researchers’ background and personal experiences, several strategies were undertaken to minimize the impact of subjective biases on the findings. For example, the researchers maintained reflective journals throughout the study, documenting their thoughts, feelings, and potential biases at each stage of the research process, and regular team discussions were held to critically examine how the researchers’ backgrounds and experiences might shape data collection, analysis, and interpretation.

### Ethical consideration

#### Ethical approval

of this study was received by the Fujian Medical University Biomedical Research Ethics Review Committee (2023 Fuyi Ethics Review No. 145). All procedures were carried out in line with the principles of the Declaration of Helsinki. Verbal informed consents were obtained from all participants prior to the commencement of the study. Participants were informed that engagement in this study is totally voluntary, that the potential risks associated with this study are minimal, and that if they feel uncomfortable during the process, they have right to withdraw from the study at any point.

## Results

This study aimed to explore the teachers’ experiences and perspectives of rehabilitation inclusion for working with deaf and hard of hearing children in rural areas. The results were presented with an overview of participants’ demographic characteristics, followed by narrating the five key themes emerged from the data. After the nineteenth interview, data saturation was deemed achieved. Among the 19 participants included in the study, two were male (10.53%) and 17 were female (89.47%). Additional demographic details were presented in Table 1. Five themes emerged from the collected data analysis: (1) role as a life-and-study co-instructor, (2) teacher-and-student bidirectional social, emotional, and financial support, (3) diverse and inclusive communication between teachers and deaf and hard of hearing children, (4) lack of policy-related, institutional and cultural information about deaf and hard of hearing children, and (5) awareness the rehabilitation to be integrated within the educational context.

**Table 1:** Basic characteristics of included participants

Variables	Included participants
Age group, number (%)	
20–29 years	3 (15.79%)
30–39 years	11 (57.89%)
≥ 40 years	5 (26.32%)
Length of teaching, number (%)	
< 5 years	12 (63.16%)
≥ 5 years	7 (36.84%)
Sex, number (%)	
Male	2 (10.53%)
Female	17 (89.47%)
Educational level, number (%)	
Above high school	14 (73.68%)
High school or equivalent	5 (26.32%)

### Theme 1: role as a life-and-study co-instructor

Due to the unique nature of the school and the specific characteristics of students with DHH, many participants reported that their role extended beyond that of a traditional teacher. In the classroom, they served as teachers responsible for imparting knowledge, while they took on the role of life instructors outside of class to build supportive and caring relationships with students. In this regard, the role could be defined as a life-and-study co-instructor to fully describe the breadth of their perceived responsibilities. When discussing their experiences with DHH students, participants shared the following:

“*As a teacher responsible for this class*,* I spent nearly six years with my students from grade one to grade six. During this period*,* I cared not only about teaching academic content*,* but also about life-related details. For example*,* I paid attention to whether they were well-fed*,* well-dressed*,* or being bullied.” (P3*,* five years of teaching)*.

*“I remember when the students first came to this school*,* they were filled with fear of the unfamiliar environment*,* unfamiliar people*,* unfamiliar buildings*,* and unfamiliar companions. It was at that moment I realized the importance of providing them not only with academic instruction but also with emotional support and compassion. In instances where one door closes*,* it is imperative for educators to strive to open another door for their students*,* thereby expanding their horizons and alleviating feelings of isolation and despondency.” (P8*,* four years of teaching)*.

*“The majority of the children come from remote mountainous regions where parental presence is often lacking. Additionally*,* due to congenital disabilities such as deafness*,* they encounter barriers to educational opportunities compared to their peers without such impairments. Given the opportunity to enroll in this school*,* it is imperative that we not only provide academic support but also extend compassionate care to enhance their quality of life and foster a sense of love and belonging.” (P1*,* two years of teaching)*.

### Theme 2: teacher-and-student bidirectional social, emotional, and financial support

The support between teachers and students is mutual. For teachers, receiving affirmation from their students strengthens their dedication to their chosen profession. While the majority of teachers acknowledged that they faced challenges in achieving the same opportunities as those working in regular schools, they found deep satisfaction in their endeavors to provide deaf and hard of hearing students with equitable educational opportunities. These efforts not only empowered DHH students but also enabled them to develop a degree of competitiveness in society.

*“When it comes to various teaching competitions or honorary awards*,* we (referring to teachers working with DHH students) often find ourselves at a disadvantage compared to our counterparts in general education due to limited resources and opportunities. As a result*,* it is challenging for us to achieve significant breakthroughs……However*,* my perseverance can be attributed to the encouragement and recognition I receive from my students.” (P11*,* five years of teaching)*.

*“Perhaps the initial goals may differ*,* but I believe that our underlying intentions remain the same — rooted in love for these children and a hope that they can access relatively equitable education opportunities……Through continuous contact and interaction*,* we become immersed in their world while also receiving affirmation from them.” (P2*,* two years of teaching)*.

In addition, working with DHH students appears to be an ideal occupation in an era marked by involution. On the premise of ensuring a fulfilling life, they have learned to slow down their pace. This is because what these students truly require is their unwavering patience and perseverance in orienting them. For example, “I find myself repeatedly pronouncing new vocabulary to aid their memorization and articulation. Simultaneously, I also need to utilize various forms of body language to stimulate their enthusiasm and maintain their attention…… Whenever I feel like giving up, they never fail to surprise us. Through this continuous dedication, I have come to experience a profound sense of belonging and professional identity.” (P9, four years of teaching).

For the students, they receive not only academic instruction, but also nurturing care akin to a mother’s love. As one teacher expressed, “I often engage in conversations with students after class. They are eager to share their stories with me, and I am willing to listen and offer my insights in return. This support is mutual — I am not only a listener but also a storyteller.” (P3, five years of teaching)

*“I understand that students at this age enjoy snacking*,* so I often buy some snacks and place them on the table. Sometimes*,* they initiate conversations with me*,* and we talk while enjoying the snacks together. It seems that language has the power to transcend their hearing impairments…… On occasion*,* I also share my stories with them*,* and they listen attentively. Although our communication isn’t always seamless*,* we genuinely cherish and respect each other.” (P7*,* six years of teaching)*.

*“As the weather grows colder*,* I often bring some outerwear from my own children for them to wear*,* hoping to shield them from the chill and prevent potential illnesses…… To be honest*,* these mutual bonds often form in such subtle*,* unintentional moments*,* and continue to deepen and strengthen over time.” (P19*,* two years of teaching)*.

### Theme 3: diverse and inclusive communication between teachers and deaf and hard of hearing children

Given the diverse backgrounds of students with DHH, their sign languages naturally exhibit nuanced variations. Without a period for adaptation and interaction, minor misinterpretations can arise when attempting to comprehend one another’s sign language. As such, teachers incorporate body language or articulate their thoughts through written communication when engaging with students with DHH. As one teacher elaborated: “Chinese sign language has regional characteristics, meaning that the sign language from one region is incompatible with another. In such cases, depending exclusively on sign language for communication is challenging and time-consuming. Occasionally, for simplicity, we opt to type out our messages. However, this method is more accessible to older students. Younger students may struggle to write certain words due to their limited vocabulary. In these situations, it becomes crucial for us to patiently interpret the meaning behind their sign language expressions in order to facilitate effective communication.” (P12, three years of teaching)

*“Sometimes when students communicate with us via WeChat*,* the word order in their messages is often inverted. They tend to express their thoughts or reflections directly instead of structuring their sentences in the subject-verb-object format that we typically use.” (P4*,* two years of teaching)*.

In addition to the diversity of communication and interaction styles, we, as teachers, actively promoted the use of inclusive language in our interactions with students. This approach ensured that they felt valued as integral members of our community, thereby fostering a stronger sense of belonging and inclusion.

*“Each student has a distinct communication style along with individual preferences and habits regarding interpersonal interactions. I firmly believe that*,* as a teacher*,* I should not impose a communication mode solely for my convenience. Instead*,* I should equally respect each student’s preferred language and interaction style.” (P10*,* eight years of teaching)*.

*“In every interaction with my students*,* I often begin by quoting their previous statements. For example*,* I might say*,* ‘I noticed you mentioned that…’ before articulating my own perspectives. If there are parts of the conversation that I didn’t hear clearly*,* I jot them down and ask for clarification after they finish speaking. For instance*,* I might ask*,* ‘Regarding what you just said… did you mean…?’ Through this approach*,* I aim to make them feel respected and more willing to engage in further dialogue*,* thus establishing a virtuous cycle of communication.” (P5*,* six years of teaching)*.

*“When communicating with students*,* I regard them as normal individual. We have simply adjusted our communication strategies while maintaining consistent thoughts and behaviors.” (P13*,* four years of teaching)*.

### Theme 4: lack of policy-related, institutional and cultural information about deaf and hard of hearing children

A comprehensive understanding of policies related to the rights and welfare of students with DHH can serve as an invaluable resource for these students and their families. However, when questioned about such information, most teachers were only familiar with the welfare initiatives and overarching policies specific to their own schools. They lacked a thorough and systematic understanding of relevant information that could be utilized to support both students and parents as needed.

*“What I currently know includes policies such as the ‘Two Frees and One Subsidy’ program*,* which encompasses exemption from tuition*,* accommodation*,* book fees*,* and other educational expenses*,* as well as the provision of a living allowance……Furthermore*,* additional services*,* such as hearing screenings*,* are also available to support these individuals.” (P7*,* six years of teaching)*.

*“If students meet the criteria*,* existing policies offer free cochlear implant surgeries. Unfortunately*,* many parents are unaware of this valuable information and*,* consequently*,* often miss the optimal window for implantation.” (P18*,* five years of teaching)*.

*“In addition to receiving government support*,* we have also benefited from numerous donations from compassionate organizations and individuals. Their generous contributions further aid underprivileged students.” (P6*,* four years of teaching)*.

*“Currently*,* policy initiatives aimed at promoting inclusive education and employment opportunities for students with DHH are insufficient. After completing their education here*,* these students frequently encounter significant challenges in securing employment. It is imperative that future policies prioritize addressing these issues to provide stronger support for this vulnerable group.” (P15*,* three years of teaching)*.

*“In fact*,* when it comes to policies*,* I am familiar with certain programmatic and overarching documents. However*,* my knowledge of or involvement with other local or organizational policies may be limited.” (P1*,* two years of teaching)*.

When asked about the cultural aspects related to students with DHH, most teachers expressed confusion, possibly due to the lack of discourse on culturally relevant factors within our country. As one teacher explained, “In terms of culture, I am unsure about the specific culture you are referring to. From my perspective, it should encompass Chinese culture. I believe that these DHH students embody virtues rooted in traditional Chinese culture, such as resilience.” (P14, five years of teaching).

### Theme 5: awareness the rehabilitation to be integrated within the educational context

As rehabilitation concepts continue to evolve in China, a growing number of individuals are beginning to understand and participate in rehabilitation practices. For students with DHH, early rehabilitation can significantly influence their future development and quality of life. When queried about their understanding of rehabilitation strategies, teachers commonly emphasized the importance of early rehabilitation and the need to educate parents about rehabilitative knowledge and concepts. This ensured that students could benefit from timely interventions.

*“In terms of rehabilitation*,* my current understanding focuses primarily on speech therapy for these students. Our school offers a tailored speech training program specifically designed to assist them during the initial stages of speech rehabilitation*,* ensuring that they receive timely and effective training at the optimal time.” (P16*,* three years of teaching)*.

*“Regarding rehabilitation*,* cochlear implants should indeed be recognized as therapeutic interventions. Regrettably*,* many students tend to utilize them at a later stage*,* thereby missing the optimal period for maximizing the rehabilitation benefits these devices can provide.” (P9*,* four years of teaching)*.

*“I often read relevant articles on public platforms*,* and I recall coming across a piece advocating for sensory integration training as an effective rehabilitation strategy for these students…… Additionally*,* there are rehabilitation centers or institutions in society that offer such services. However*,* this requires parental cooperation and sufficient financial support.” (P17*,* three years of teaching)*.

Students with DHH have distinct rehabilitation needs. However, there is a need to improve and refine the delivery of these services to better meet their requirements.

*“Currently*,* our school’s only resource for rehabilitation consists of speech therapists*,* with only one or two teachers involved in this role. To adequately meet the rehabilitation needs of students with DHH and other students with special needs*,* it would be beneficial to recruit additional professionals specializing in rehabilitation.” (P5*,* six years of teaching)*.

*“Rehabilitation is very important*,* but there remains a significant gap in services available for young children……Moreover*,* awareness of the need to rehabilitate children with DHH among schools*,* parents*,* and society is alarmingly low.” (P6*,* four years of teaching)*.

*“Actually*,* we can explore more models that integrate medical interventions with educational frameworks*,* so that students with DHH can receive comprehensive rehabilitation while simultaneously benefiting from equal educational opportunities.” (P19*,* two years of teaching)*.

## Discussion

The study explored teachers’ experiences and perspectives on the inclusion of rehabilitation practices when working with deal and hard of hearing children in a specialized educational setting in China. Consistent with previous studies [10, 24], the study identifies several recurring findings, including teachers’ holistic roles and responsibilities, communication barriers and the need for standardization, challenges in professional competencies and training needs, and lack of policy and institutional support. However, the study also revealed novel insights that distinguished it from prior research. First, it emphasized a shift from a unidirectional to a bidirectional relationship between teachers and students with DHH, where both parties mutually benefit from emotional, social, and academic support. This finding underscores the reciprocal nature of teacher-student interactions, which not only enhances student outcomes but also reinforces teachers’ motivation and professional identity. Second, the study highlighted the critical importance of integrating rehabilitation practices, such as speech therapy and sensory integration training, within the educational framework. This integration represents a progressive approach to addressing the unique needs of DHH students, ensuring that rehabilitation and education are not treated as separate domains but as interconnected components of a holistic support system.

The life-and-study co-instructor role perceived by teachers was similar as the parental one. As parents of children with DHH, parents may experience stress and anxiety as their children with disability transit to school [25]. Likewise, parents of children receiving special education services possessed significant concerns about their children’s behavior, communication, and academic readiness in comparison to parents of regular education students [26]. Besides, children with DHH confronted difficulties and challenges in school-based learning, such as making friends. “A sense of self” was the living characteristics of these children, which conflicted with either part of the hearing community or part of the deaf community [27]. Therefore, teachers, mimicking as children’ parent in the special school where children were totally isolated in the collective context, naturally played dual roles as life-and-study co-instructors to make children with DHH feel “a sense of belonging” rather than “a sense of self”.

Social support provided for teachers was primarily the emotional social support defined as something attached with caring, love, empathy, sympathy, understanding, and esteem delivered by significant others [28]. Another type of social support is practical social support or instrumental support, which refers to the demonstrations of advice and information as well as touchable assistance, including but not limited to physical devices, transportation, services, and materials [29]. In addition to the emotional social support already received by teachers, practical social support is also needed to obtain the “fit” social support. A high overlap of “fit” is required between what a person needs and what is provided, which can be facilitated through empathetic understanding in accordance with both social and experiential resemblances with the person in need [30]. Thus, social support at school, especially in the special educational school, is of great importance for children with DHH to impact their motivation, academic success, and overall well-being within the school environment.

The diverse sign languages of students with DHH challenged the communication and interaction between teachers and students. This finding aligned with the work of Manga and Masuku, who highlighted that the diversity among deaf-blind learners creates challenged for educators, particularly in terms of the type and level of sign language exposure, the level of family support, and the ways students engage during adolescence [8]. Chinese sign language (CSL) is the primary sign language used by the Deaf community in China, but it is not uniform across the country [31]. Regional dialects and variations in CSL can lead to misunderstandings, even among native signers. For instance, signs used in northern China may differ significantly from those used in southern China, creating challenges for teachers who work with students from diverse geographical backgrounds. This diversity mirrors the linguistic variations found in spoken Chinese dialects, such as Mandarin and Cantonese, but poses unique challenges in educational settings where standardized communication is essential [32]. In our study, teachers emphasized the importance of using alternative communication strategies to facilitate interaction with DHH students, such as written communication, body language and visual aids, speech-to-text software, etc. Furthermore, alternative strategy was proposed by teachers that the sign languages should be unified across regions in China to reduce or eliminate regional disparities. However, implementing such a strategy would require sustained efforts and collaboration with relevant administrative departments to achieve widespread adoption. Practically speaking, training teachers on DHH communication strategies is more achievable as for misinterpretations between the two parties can be prevented by facilitating an effective way of communication [33].

Rehabilitation for students with DHH, particularly early rehabilitation plays a pivotal role in shaping their life courses. However, early recognition and timely referral for diagnosis remain significant challenges among preschool students with suspected hearing impairments. These challenges are largely or partly attributed to a lack of awareness about children-related deafness and a reluctance to seek appropriate therapeutic interventions [34]. The degrees of hearing loss, ranging from mild to profound, significantly influenced the choice of interventions and rehabilitation strategies for children with DHH. The study consisting of 51 children with mild-to-profound hearing loss and 37 children with normal hearing demonstrated that children with an average hearing loss of 70 dB HL or better generally achieved language skill in line with those of age-matched norms. In contrast, children with severe and profound hearing loss exhibited significantly more variability in their language outcomes [35]. A study revealed that hearing aid use and auditory habilitation with auditory-verbal therapy significantly improved acoustic voice parameters of children with bilateral profound hearing loss [36]. Regardless of degree of hearing loss, children with early enrollment in interventions achieved better language scores [37]. A growing body of studies showed that earlier implantation of cochlear implants (CI) contributed to a speedier rate of vocabulary growth, higher expressive spoken language scores, and higher performance levels on reading assessments [38–41]. Furthermore, early access to sign language for CI users with late implantation can serve as a scaffold for appropriate cognitive development, while early access to oral language for those with early implantation further improved cognitive outcomes [42]. As such, it is imperative to provide personalized interventions tailored to the specific needs of individuals based on their level of hearing loss, as this approach is critical for maximizing communication, language development, and overall quality of life.

Although this study may shed light on the reforms and developments in special education and arouse public awareness of the dual teachers-and-students’ needs in this educational context, there were several limitations existing in our study. First, the sample size was relatively small due to the nature of qualitative research design. Second, participants recruited in this study were from a public special education school located in Jian’ou, Fujian province, China, which may impede the generalizability to wider populations. Finally, the study would benefit from the inclusion of quantitative data to further strengthen the evidence.

## Conclusion

In conclusion, this study extracted five key themes based on the textual data, including (1) role as a life-and-study co-instructor, (2) teacher-and-student bidirectional social, emotional, and financial support, (3) diverse and inclusive communication between teachers and deaf and hard of hearing children, (4) lack of policy-related, institutional and cultural information about deaf and hard of hearing children, and (5) awareness the rehabilitation to be integrated within the educational context. Based on the study’s findings, the following recommendations are proposed: First, a standardized Chinese Sign Language curriculum should be developed to reduce regional disparities. Second, policy initiatives should be implemented to improve access to hearing screenings, hearing aids, cochlear implants, and sign language instruction, particularly in rural and underserved areas in China. Third, it is essential to increase funding and allocate more resources to public special education schools in underdeveloped rural areas, ensuring that both teachers and students have access to the necessary support, infrastructure, and opportunities for growth. Lastly and importantly, public awareness about Deaf culture should be actively raised to promote a deeper understanding and acceptance of sign language and Deaf identity within schools and the broader community. Although this study provides valuable insights into the experiences and needs of the targeted population, future research should incorporate rigorous quantitative methods to strengthen the scientific foundation of the findings and enhance their generalizability.

## Electronic supplementary material

Below is the link to the electronic supplementary material.

Supplementary Material 1

## Data Availability

The datasets that support the findings are available from the corresponding author or first author upon reasonable request.

## Abbreviations

DHH: Deaf and Hard of Hearing
WHO: World Health Organization
COREQ: Consolidated Criteria for Reporting Qualitative Research
CI: Cochlear Implants
CSL: Chinese Sign Language
