# Supplementary material for: “It’s beyond enough”: teachers’ experiences and perspectives of rehabilitation inclusion for working with deaf and hard of hearing children in rural areas of China
Source: BMC Public Health. 2025 May 19;25:1841. doi: 10.1186/s12889-025-22972-1 (PMC12087191; doi:10.1186/s12889-025-22972-1)
Supplement: Supplementary file 1 — Supplementary Material 1 [file 12889_2025_22972_MOESM1_ESM.docx]

**SEMI-STRUCTURED INTERVIEW QUESTIONNAIRE FOR
TEACHERS WORKING WITH DEAF AND HARD OF HEARING CHILDREN**

### **Questionnaire guides**

These questions were aimed to facilitate discussion but do not need to be asked in the sequence listed below. The interviewer should be prepared to follow up on any additional issues that may emerge and seem significant in relation to the aforementioned issues. The questions are formulated in full to ensure they are open-ended and non-directive, and cover all the issues we need to explore. However, you can adjust the wording to make it more conversational, but make sure you cover the content and refrain from leading the participant. We all possess unconscious bias and assumptions and ensure that these aren’t reflected in the following questions.

### **Conducting the interviews**

**Introduction:** Good morning/afternoon, my name is XXX. Thank you for speaking with me today. I’d like to talk to you about your experiences and perception of rehabilitation inclusion for working with deaf and hard of hearing children in your school.

**Reminder:** The confidentiality and anonymity should be explained in detail and obtain the verbal consent before initiating the interviews. Reiterate that we will be asking questions about their experiences as a teachers working with deaf and hard of hearing children. What you share with us will be invaluable. If there are any questions you don’t want to answer, you don’t have to – that’s OK. Remind them that if you feel uncomfortable during the process, you have right to withdraw from the study at any point.

***Semi-structured interview questionnaire***

(1) Could you tell me about your experiences with deaf and hard of hearing children?

(2) How do you communicate with deaf and hard of hearing children?

(3) Could you tell me about your relationship with deaf and hard of hearing children? And how to maintain a good relationship with them?

(4) How do you collaborate with other teachers for working with deaf and hard of hearing children? And what is your role in it?

(5) Could you talk about some policy-related, cultural, and institutional information for deaf and hard of hearing children?

(6) Could you talk about the impact of working with deaf and hard of hearing children?

(7) As a deaf children education worker, could you talk what support you need? And what support is already available? What is to be gained?

(8) Could you talk about the barriers encountered as an educator of deaf and hard of hearing children? And how do you think these barriers can be effectively addressed?

(9) Could you talk about your understanding of rehabilitation?

(10) What rehabilitation-related needs do you think exist for deaf and hard of hearing children?

(11) Could you talk about the rehabilitation-related personnel, equipment, and services available at your current school/institution? What do you think needs to be further strengthened or improved?

(12) Is there anything else you'd like to share with us?

***Closing***

Thank you for your time and information provided today. Hope you have a good day.
